# Synergistic Effects of Heat-Treated Green Tea Extract and Enzymatically-Modified Isoquercitrin in Preventing Obesity

**DOI:** 10.3390/nu15132931

**Published:** 2023-06-28

**Authors:** Ye-jin Moon, Hee-seong Kim, Min-ji Kim, Hyeon-yeong Im, Yun-hee Lee

**Affiliations:** College of Pharmacy and Research Institute of Pharmaceutical Sciences, Seoul National University, Seoul 08826, Republic of Korea; moon4wy@snu.ac.kr (Y.-j.M.); 99dkfqkq@snu.ac.kr (H.-s.K.); kminji1222@snu.ac.kr (M.-j.K.); hy1210@snu.ac.kr (H.-y.I.)

**Keywords:** obesity, adipose tissue, lipolysis, browning, PKA signaling, heat-treated green tea extract, enzymatically modified isoquercitrin

## Abstract

Previous research has shown that both heat-treated green tea extract (HTGT) and enzymatically modified isoquercitrin (EMIQ) have anti-obesity effects. Given the absence of in vivo evidence demonstrating their synergistic effects, our study aimed to elucidate the combined obesity prevention potential of HTGT and EMIQ in mice. Mice were treated with these compounds for 8 weeks, while being fed a high-fat diet, to investigate their preventive anti-obesity effects. We demonstrated that the co-treatment of HTGT and EMIQ results in a synergistic anti-obesity effect, as determined by a Kruskal–Wallis test. Furthermore, the combined treatment of HTGT and EMIQ was more effective than orlistat in reducing body weight gain and adipocyte hypertrophy induced by high-fat diet. The co-treatment also significantly reduced total body fat mass and abdominal fat volume. Additionally, the group receiving the co-treatment exhibited increased energy expenditure and higher glucose intolerance. We observed a dose-dependent upregulation of genes associated with mitochondrial oxidative metabolism and PKA signaling, which is linked to lipolysis, in response to the co-treatment. The co-treatment group displayed elevated cAMP levels and AMPK activation in adipose tissue and increased excretion of fecal lipids. The results indicate that the co-treatment of HTGT and EMIQ holds the potential to be a promising combination therapy for combating obesity. To further validate the anti-obesity effect of the combined treatment of HTGT and EMIQ in human subjects, additional clinical studies are warranted.

## 1. Introduction

Obesity poses substantial implications for public health, as it is associated with an elevated risk of chronic diseases, including diabetes and cardiovascular disorders [1]. Obesity is primarily caused by an imbalance between the intake and expenditure of energy, where excess calories are stored in the body as fat [2]. The existing treatments for obesity encounter challenges regarding long-term effectiveness and sustainability, making it difficult for individuals to achieve and maintain weight loss [3]. Considering the potential adverse effects associated with current pharmaceutical interventions for obesity treatment, our study focuses on investigating the potential of phytochemicals as dietary supplements, offering promising alternatives for obesity prevention. The prevalence of obesity has been steadily rising in the recent few years, highlighting the need for effective strategies to prevent and treat this public health problem.

Adipose tissue exerts a crucial function in energy metabolism and is composed of adipocytes, which are specialized cells that store energy in lipid droplets [4]. Adipose tissue is generally classified into two major categories: brown adipose tissue (BAT) and white adipose tissue (WAT) [5]. BAT is characterized by its high mitochondrial content and uncoupling protein 1 (UCP1) expression, which is important for non-shivering thermogenesis [6]. Conversely, WAT serves as a site for lipid storage in the form of triglycerides (TG), which can be utilized during periods of energy demand [7]. WAT can acquire BAT-like properties through cold exposure or β-adrenergic signaling, which is a process termed browning or beiging [8]. Enhancing energy expenditure through the activation of BAT and browning of WAT represents a promising strategy for treating obesity and related metabolic diseases. The protein kinase A (PKA) signaling pathway is a key player in BAT activation and WAT browning, as it serves as a major regulator of cytosolic lipolysis by activating hormone-sensitive lipase (HSL) and promoting subsequent mitochondrial oxidative metabolism [9,10].

Epigallocatechin gallate (EGCG) is a prominent phytochemical found in green tea extract, and approximately half of EGCG undergoes epimerization to form gallocatechin gallate (GCG) during the heat treatment process [11]. A previous study demonstrated the effects of heat-treated green tea extract (HTGT) on visceral fat and hepatic triacylglycerol in rats [12], while our group confirmed that HTGT induces significantly higher expression of UCP1 in brown adipocytes, compared to green tea extract (GTE), due to its elevated content of GCG [13]. Enzymatically modified isoquercitrin (EMIQ) refers to the enzymatic derivatives of rutin, where glucose-rhamnose is attached to the position 3 of the quercetin glycosides [14,15]. This modification enhances the bioavailability of EMIQ, making it more effective compared to quercetin alone [16]. EMIQ treatment enhanced energy metabolism mainly by phosphorylating 5′ adenosine monophosphate-activated protein kinase (AMPK) in WAT [17].

Our previous study further demonstrated the synergistic anti-obesity effect achieved through the activation of adipose tissue metabolism, when combining EMIQ with soybean embryo extract [18]. In our previous study, we reported the anti-obesity effects of the combined treatment of HTGT and EMIQ. However, previous studies primarily focused on the short-term effects of the HTGT and EMIQ combination in terms of anti-obesity properties [13]. Furthermore, investigations into their in vivo synergistic effects and molecular mechanisms have been limited. Therefore, this study aims to examine the long-term synergistic anti-obesity effects of HTGT and EMIQ, while exploring their underlying molecular mechanisms. Given that HTGT and EMIQ activate two distinct pathways, we hypothesized that their combined administration would demonstrate a synergistic effect in terms of anti-obesity properties. To examine their preventive anti-obesity effects, mice were treated with the compound continuously during a high-fat diet (HFD) feeding regiment, in contrast to our previous study where obese mice were treated with the compounds after 8 weeks of HFD feeding. During the course of the study, HTGT and EMIQ were administered orally, either individually or in combination, at different doses (100 or 200 mg/kg), for a duration of 8 weeks. Additionally, a positive control group was included, which received orlistat, an approved anti-obesity drug currently available in the market. This study aimed to elucidate the long-term synergistic anti-obesity effects of the combination of HTGT and EMIQ, and to investigate the molecular mechanisms underlying their observed effects.

## 2. Materials and Methods

### 2.1. Materials

**Figure 1:**
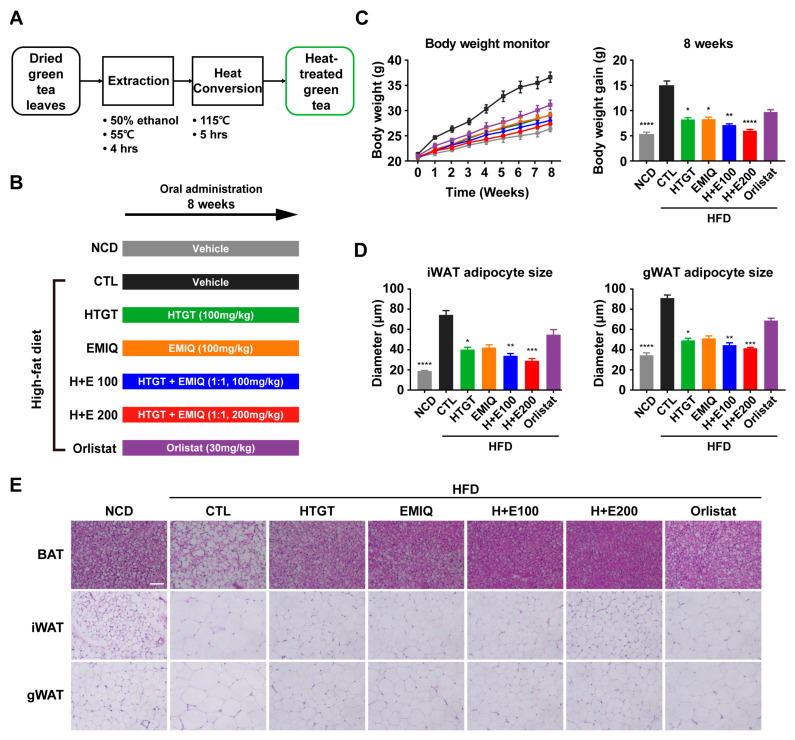
Effects of a mixture of HTGT and EMIQ on body weight gain and adipocyte size of HFD-fed mice. (**A**) Schematic diagram of heat-treated green tea production; (**B**) Experimental design while 8 weeks of NCD or HFD-fed mice treated with vehicle control, HTGT (100 mg/kg), EMIQ (100 mg/kg), H + E100 (HTGT 50 mg/kg + EMIQ 50 mg/kg), H + E200 (HTGT 100 mg/kg + EMIQ 100 mg/kg) and orlistat (30 mg/kg); (**C**) Body weight monitoring and body weight gain. NCD (gray), HFD CTL (black), HFD/HTGT (green), HFD/EMIQ (orange), HFD/H+E100 (blue), HFD/H+E200 (red), HFD/Orlistat (purple); (**D**) Adipocyte size of inguinal white adipose tissue (iWAT) and gonadal white adipose tissue (gWAT); (**E**) H&E staining of paraffin sections of adipose tissues. Size bar = 50 μm. Statistical significance was assessed using the Kruskal–Wallis test. The data are presented as means ± SEM. *n* = 6. * *p* < 0.05; ** *p* < 0.01; *** *p* < 0.001; **** *p* < 0.0001.

HTGT (Lot #. 180426) was made according to the previous description. In short, dried green tea leaves underwent extraction using 50% ethanol, followed by heat treatment at 115 °C for 5 h. After heating, EGCG in green tea was converted to GCG through epimerization. Figure 1A displays the flow chart illustrating the heat treatment processing procedures, while Table S1 presents the chemical constituents before and after the heat treatment. EMIQ (Lot #. 20191102) was made by hydrolyzing rutin and then treating with glycosyltransferase to transglycosylation [19]. HTGT and EMIQ were supplied by Amorepacific Corp. (Seoul, Republic of Korea) and orlistat was purchased from Sigma (St. Louis, MO, USA).

### 2.2. Animals

C57BL/6 mice (male, 8 weeks) were purchased from Joongah Bio (Korea), and the protocols used for the animal study were approved by Institutional Animal Care and Use Committees of Seoul National University (SNU-220209-1-3). All animal experiments were conducted at Animal Center for Pharmaceutical Research in Seoul National University, and mice were kept under normal conditions with free access to diet and water (22 ± 1 °C, 12/12 h light-dark cycle, humidity of 50 ± 5%).

Mice were divided into seven groups randomly: (i) normal chow diet (NCD), (ii) high-fat diet (HFD CTL), (iii) HFD + orlistat (HFD/Orlistat), (iv) HFD + HTGT (HFD/HTGT), (v) HFD + EMIQ (HFD/EMIQ), (vi) HFD + low-dose of HTGT and EMIQ (HFD/H + E100), and (vii) HFD + high-dose of HTGT and EMIQ (HFD/H + E200). Mice were continuously provided with a 60% high-fat diet (#D12492, Research Diets) for a duration of 8 weeks, except for the NCD group. During the 8 weeks, orlistat (30 mg/kg), HTGT (100 mg/kg), EMIQ (100 mg/kg), low-dose of the mixture of HTGT and EMIQ (50 + 50 mg/kg), or high-dose of the mixture of HTGT and EMIQ (100 + 100 mg/kg) was orally administered to the mice once a day. All the materials were dissolved in distilled water; NCD and HFD CTL groups were orally treated with equal volumes of distilled water as the vehicle. The body weight was recorded weekly. All mice were euthanized by CO_2_ inhalation.

### 2.3. Cell Culture

C3H10T1/2 (ATCC, CCL-266, Manassas, VA, USA) cells were cultured at 37 °C with 5% CO_2_. Dulbecco’s modified eagle medium (DMEM, Welgene, Gyeongsan-si, Republic of Korea) was used and supplemented with 10% fetal bovine serum (FBS, Gibco, Seoul, Republic of Korea) with 1% penicillin/streptomycin (P/S, Welgene). To induce fully differentiated C3H10T1/2 adipocytes, confluent cells were exposed to 20 ng/mL BMP4 for 2 days, and differentiation medium containing 0.5 mM IBMX, 1 μM dexamethasone, 10 μg/mL insulin, 0.125 mM indomethacin and 1 nM triiodothyronine (T3) for three days. After the induction of differentiation, cells were maintained in medium with 10 μg/mL insulin and 1 nM T3 for 3 days. For in vitro adipocyte experiments, differentiated C3H10T1/2 adipocytes were treated with a vehicle control, the mixture of HTGT and EMIQ (100 μg/mL), Compound C (10 μM), or H 89 dihydrochloride (50 μM) for indicated times.

### 2.4. Body Composition Analysis and Micro-Computer Tomography (Micro-CT)

The body composition analyses of mice were conducted using a nuclear magnetic resonance (NMR) technology system with EchoMRI-700 (Echo Medical Systems, Nanded, India). The abdominal regions of mice were scanned by Quantum GX2 micro-CT imaging system (PerkinElmer, Waltham, MA, USA) following the published method with some modifications [20,21]. Briefly, mice were anesthetized in the chamber using isoflurane, and then placed on the CT bed, to which, the inhalation anesthesia machine was connected. The X-ray tube was operated at 90 kV/88 μA, the field of view (FOV) was 45mm, and the size of pixel was 90μm. The scan mode was set to 4 min with high resolution. The volumes of abdominal fat regions between the proximal end of the L1 vertebrae and the distal end of the L5 vertebrae were calculated using AnalyzeDirect software (ver. 12.0, Overland Park, KS, USA).

### 2.5. Histology and Immunofluorescence Staining (Immunohistochemistry)

Hematoxylin and eosin (H&E) staining was conducted [22]. To measure the diameters of adipocytes, images obtained by 20× magnifying the H&E-stained paraffin sections were taken and used.

For immunohistochemistry, anti-UCP1 antibody (1:400 dilution; UCP11-A, Alpha Diagnostic International) was used to stain deparaffinized tissues at 4 °C overnight. Goat anti-rabbit Alexa Flour 488 (1:500 dilution; Thermo Fisher, Waltham, MA, USA) was used for a secondary antibody and stained for 1h at RT. DAPI (Sigma, St. Louis, MO, USA) was used to stain nuclei. Histological images were captured by LSM800 confocal microscope (Zeiss, Jena, Germany) and the adipocyte sizes were measured by Zen software (ver. 3.0).

### 2.6. Glucose Tolerance Test and Indirect Calorimetry Analysis

For the glucose tolerance test (GTT) [23], mice were administered an intraperitoneal injection of D-glucose (2 g/kg; Sigma, USA) after 12-h fasting. The glucose levels in the blood were measured at 0, 15, 30, 45, 60, 90, 120, and 150 min using test strips and a Gluco Dr.Top blood glucose meter (Allmedicus, Anyang-si, Republic of Korea).

Oxygen consumption (VO_2_), carbon dioxide production (VCO_2_), energy expenditure (EE), activity, and food intake were measured utilizing PhenoMaster (TSE system, Germany) for 48 h. During monitoring, mice were maintained at 24 °C and 12-h/12-h (light/dark) cycle while water and diet were provided ad libitum.

### 2.7. Pancreatic Lipase Activity Assay and Fecal Lipid Extraction

A pancreatic lipase activity assay was conducted, as previously mentioned [24]. The measurement of excreted lipids from feces was performed, as explained in Folch’s method with some modifications [25]. Briefly, 1000 mg of feces were collected per mouse, and lipids were extracted by the solution of chloroform and methanol (2:1 ratio, *v*/*v*). After evaporating the solution of the lipid phase for 3 days, the dried lipid was weighed and calculated.

### 2.8. Enzyme-Linked Immunosorbent Assay (ELISA)

Adiponectin, leptin, and cAMP were quantified by ELISA kit (#ab226900, #MOB00, #ADI-900-066A). All the protocols of ELISA experiments were conducted according to the manufacturer’s guidelines and protocols.

### 2.9. Quantitative Real-Time PCR (qRT-PCR)

qRT-PCR was conducted following the previously described protocol [26]. Briefly, total RNA from adipose tissues was extracted with TRIzol (Invitrogen, Waltham, MA, USA). Reverse transcription of RNA into cDNA was carried out using cDNA Reverse Transcription kit (Applied Biosystems, Waltham, MA, USA). PCR amplification was performed using iQ SYBR Green Supermix on CFX Connect Real-time system (Bio-rad, Hercules, CA, USA). The primers used for qRT-PCR are listed in Table 1.

### 2.10. Western Blot Analysis

Protein from adipose tissues was extracted by protein extraction solution PRO-PREP (iNtRON Biotechnology, Seongnam-si, Korea), with PhosSTOP phosphate inhibitor (Roche, Basel, Switzerland) and SIGMAFAST Protease Inhibitor (Sigma, Livonia, MI, USA). The concentration of protein was determined by MultiSkan GO spectrophotometry (Thermo Fisher, Waltham, MA, USA) with Pierce BCA Protein Assay Kit (Thermo Fisher, Waltham, MA, USA) at 562 nm. Proteins were separated by size on the SDS-PAGE gel (8% or 12%) and transferred to polyvinylidene difluoride (PVDF) membranes (Bio-Rad, Hercules, CA, USA). The membranes were blocked for 1 h in blocking solution (5% bovine serum albumin or powdered skim milk in TBST) and then incubated with primary antibodies overnight. After washing the membranes, they were incubated with secondary antibodies (HRP-conjugated anti-mouse, anti-rabbit secondary antibodies, Thermo Fisher, Waltham, MA, USA) for 1 h. The protein bands were visualized using Fusion Solo chemiluminescence imaging system (Vilber Lourmat, Collégien, France) and EvolutionCapt program (ver. 17.03). The quantification of immunoblots was using ImageJ (National Institutes of Health, Bethesda, MD, USA). The primary antibodies were listed in Table S2.

### 2.11. Statistics

This study used GraphPad Prism 7 (ver. 7.0, GraphPad software, Boston, MA, USA) for statistical analysis. The data are presented as mean ± SEM (standard error of the mean). Statistical significance was determined using the Kruskal–Wallis test for comparisons involving more than two groups, and the Mann–Whitney test for comparisons between two groups. A *p*-value less than 0.05 was considered statistically significant.

## 3. Results

### 3.1. HTGT and EMIQ Synergistically Prevented High-Fat Diet-Induced Obesity in Mice

We investigated the synergistic obesity prevention effect of HTGT and EMIQ by administering them individually or in combination to mice over a period of 8 weeks of HFD feeding. Orlistat, an anti-obesity drug that inhibits pancreatic lipase, served as the positive control (Figure 1B). Vehicle controls of HFD-fed mice showed significant body weight gains, compared to mice on an NCD. Interestingly, the co-treatment of HTGT and EMIQ resulted in a dose-dependent reduction in body weight gain that compared to individual treatments of HTGT or EMIQ throughout the 8 weeks, and surpassed the effectiveness of orlistat. (Figure 1C). Histological analysis of iWAT and gWAT revealed a dose-dependent decrease in the cross-sectional diameters of adipocytes in the mixture groups compared to the single compound groups (Figure 1D,E).

### 3.2. HTGT and EMIQ Synergistically Reduced Overall and Abdominal Adiposity

**Figure 2:**
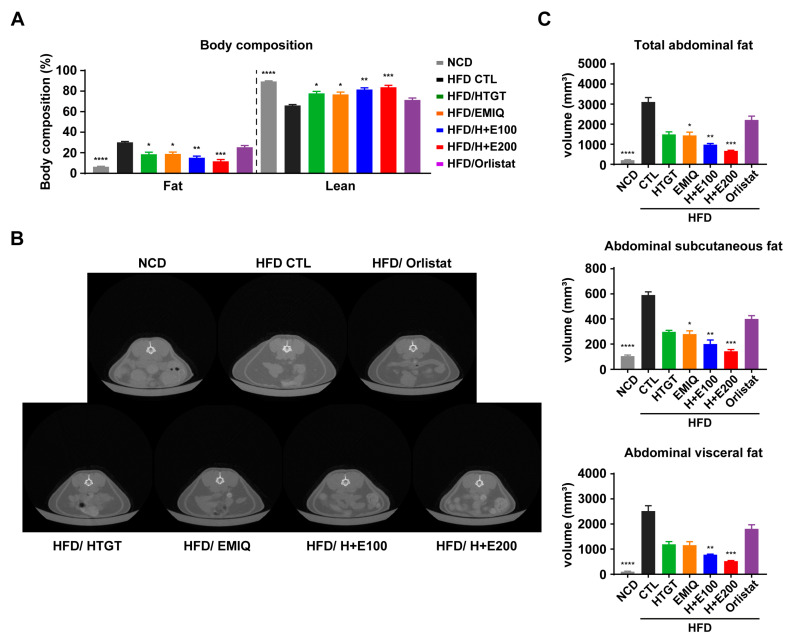
Effects of a mixture of HTGT and EMIQ on body composition and abdominal adiposity of HFD-fed mice. (**A**) Body composition of fat and lean mass of mice; (**B**) Representative transverse micro-CT images of abdominal fat between lumbar vertebrae number 1–5; (**C**) Total, subcutaneous, and visceral abdominal fat volumes of mice using micro-CT image analysis. Total abdominal fat is the sum of subcutaneous and visceral abdominal fat volume. Statistical significance was assessed using the Kruskal–Wallis test. The data are presented as means ± SEM. *n* = 6. * *p* < 0.05; ** *p* < 0.01; *** *p* < 0.001; **** *p* < 0.0001.

To evaluate the effects of co-treatment of HTGT and EMIQ against obesity, we conducted a body composition analysis. The results demonstrated a significant decrease in the percentage of fat mass and an increase in the percentage of lean mass in a synergistic and dose-dependent manner following the co-treatment (Figure 2A). Furthermore, micro-CT was utilized to assess the impact of the combination treatment on abdominal adiposity by calculating abdominal fat volume. As shown in Figure 2B, the co-treatment groups exhibited a significant reduction in both abdominal subcutaneous and visceral fat volumes in synergistic and dose-dependent manner (Figure 2C).

### 3.3. HTGT and EMIQ Synergistically Improved Glucose Tolerance and Energy Expenditure and Inhibits Pancreatic Lipase Activity

**Figure 3:**
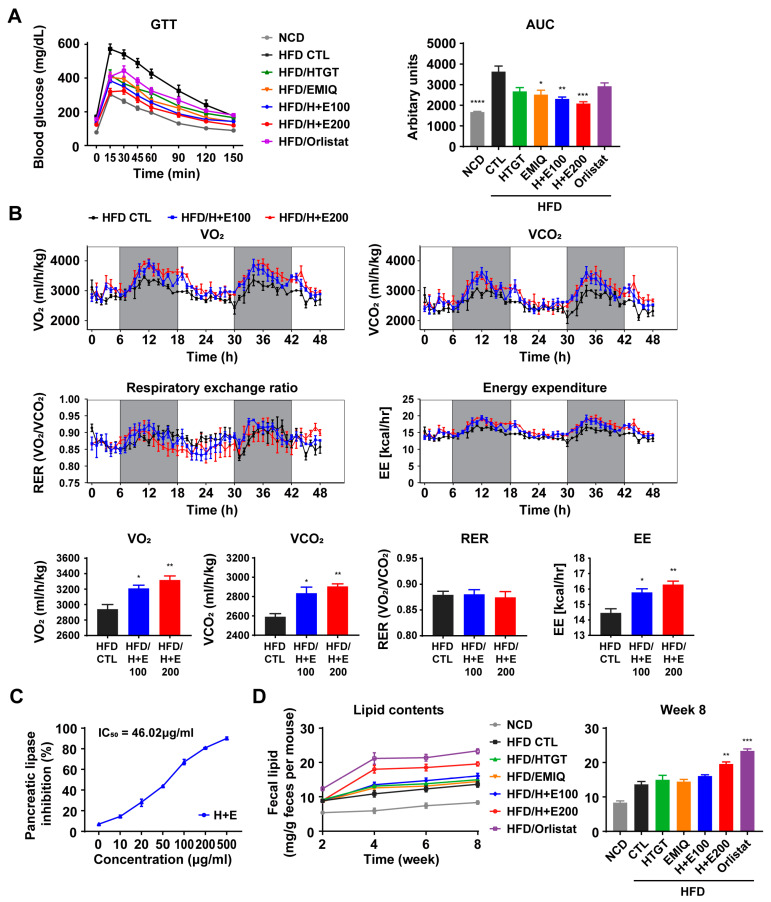
Effects of a mixture of HTGT and EMIQ on preventing HFD-induced obesity. (**A**) Intraperitoneal glucose tolerance test (GTT) of mice and area under the curve (AUC); (**B**) Indirect calorimetry analysis of vehicle-treated, low-dose of HTGT + EMIQ (100 mg/kg) and high-dose of HTGT + EMIQ (200 mg/kg)-treated HFD-fed mice. *n* = 3 per group. VO_2_ (rate of oxygen consumption), VCO_2_ (rate of carbon dioxide production), RER (respiratory exchange ratio), EE (energy expenditure); (**C**) Pancreatic lipase inhibitory activity at different concentrations of a mixture of HTGT and EMIQ; (**D**) Lipid contents in the fecal sample during 8 weeks. Statistical significance was assessed using the Kruskal–Wallis test. The data are presented as means ± SEM. *n* = 6. * *p* < 0.05; ** *p* < 0.01; *** *p* < 0.001; **** *p* < 0.0001.

Next, we evaluated the impact of the treatment on glucose tolerance by conducting an IP-glucose tolerance test. The results revealed that the co-treatment, synergistically and in a dose-dependent manner, improved glucose tolerance induced by HFD (Figure 3A). Furthermore, indirect calorimetry analysis showed that all co-treatment groups increased energy expenditure (Figure 3B).

Reduction of dietary lipid absorption by the inhibition of pancreatic lipase in the gastrointestinal tract is one of the potential mechanisms for preventing obesity. Previous studies have demonstrated the inhibitory effects of green tea and quercetin on pancreatic lipase activity [27,28]. As shown in Figure 3C, co-treatment of HTGT and EMIQ exhibited a dose-dependent inhibition of the pancreatic lipase activity with an IC_50_ of 46.02 μg/mL. In addition, we measured fecal lipid excretion in mice over the 8-week period. After 4 weeks, we observed increased lipid content in the feces of the co-treatment groups, especially in the high-dose co-treatment group (200 mg/kg) (Figure 3D).

Next, we assessed the effects of the co-treatment of HTGT and EMIQ on serum adiponectin and leptin levels. Compared to the HFD CTL group, all co-treatment groups exhibited increased serum adiponectin levels and decreased leptin levels (Figure S1). Collectively, co-treatment of HTGT and EMIQ has a synergistic effect of preventing obesity and related diseases compared to administration of the individual phytochemicals.

### 3.4. HTGT and EMIQ Synergistically Increased Mitochondrial Activity and Oxidative Metabolism in Adipose Tissues

**Figure 4:**
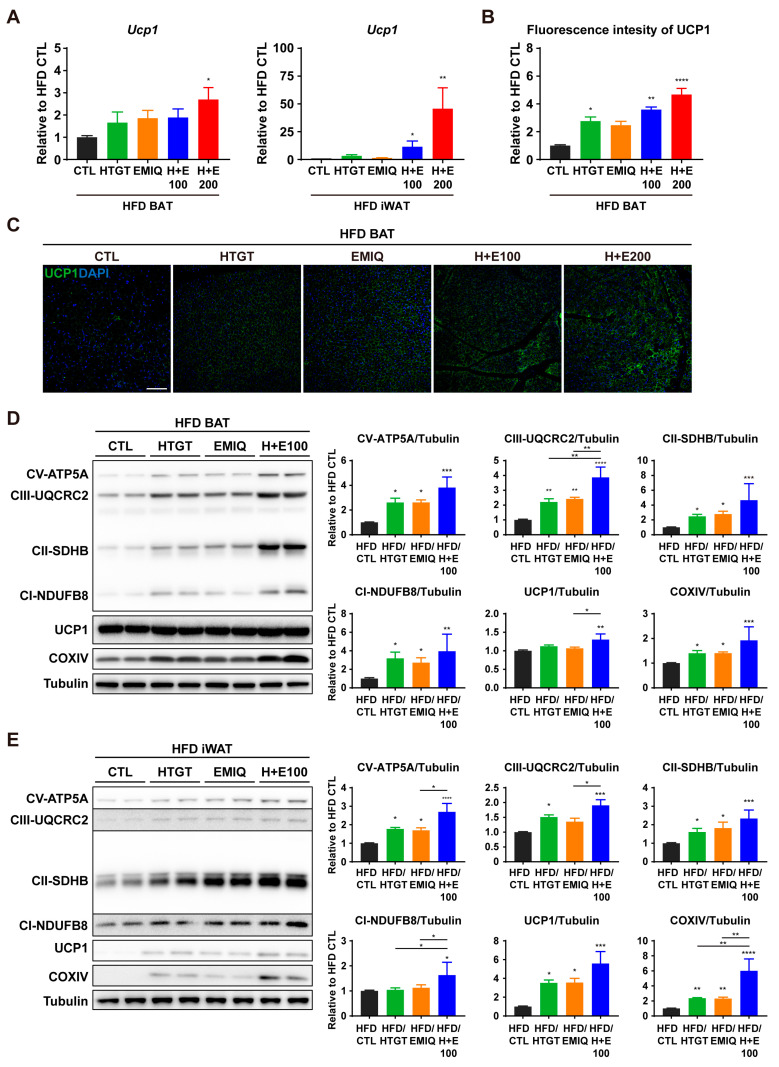
Effects of co-treatment of HTGT and EMIQ on mitochondrial metabolic activity in adipose tissues. (**A**) mRNA expression levels of UCP1 in BAT and iWAT; (**B**) Quantification of immunofluorescence intensity of UCP1 in BAT; (**C**) Representative images from immunofluorescence staining of UCP1 (green) and DAPI staining of nuclei (blue) in BAT. Size bar = 100 μm; (**D**,**E**) Immunoblot analysis and quantification of proteins involved in thermogenesis and mitochondrial oxidative metabolism in BAT (**D**) and iWAT (**E**). Statistical significance was assessed using the Kruskal–Wallis test. The data are presented as means ± SEM. *n* = 6. * *p* < 0.05; ** *p* < 0.01; *** *p* < 0.001; **** *p* < 0.0001.

To explore the potential synergism of HTGT and EMIQ on thermogenesis in adipose tissues, we confirmed the expression of UCP1, a marker of thermogenesis. The mRNA level of UCP1 in BAT tended to be higher in all treatment groups than in the HFD CTL group, but there was no statistically significant difference between administration groups. Furthermore, UCP1 mRNA expression in iWAT was higher in the co-treatment group than in other groups, but the difference in the high-dose of the co-treatment group was the only statistically significant difference (Figure 4A). As shown in Figure 4B,C, the intensity of immunofluorescence in BAT showed synergistic and dose-dependent upregulation of UCP1.

The process of adipocyte browning is tightly linked to the regulation of mitochondrial activity and oxidative capacity [29]. Therefore, protein markers related to mitochondrial metabolic activity were examined by western blot analysis. Co-treatment of HTGT and EMIQ upregulated UCP1 and several protein markers involved in mitochondrial oxidative phosphorylation in both BAT and iWAT (Figure 4D,E). These results indicate that co-treatment of HTGT and EMIQ stimulates adipocyte browning and regulation of mitochondrial activity.

### 3.5. HTGT and EMIQ Synergistically Upregulated cAMP-Dependent PKA Signaling in Adipose Tissues

**Figure 5:**
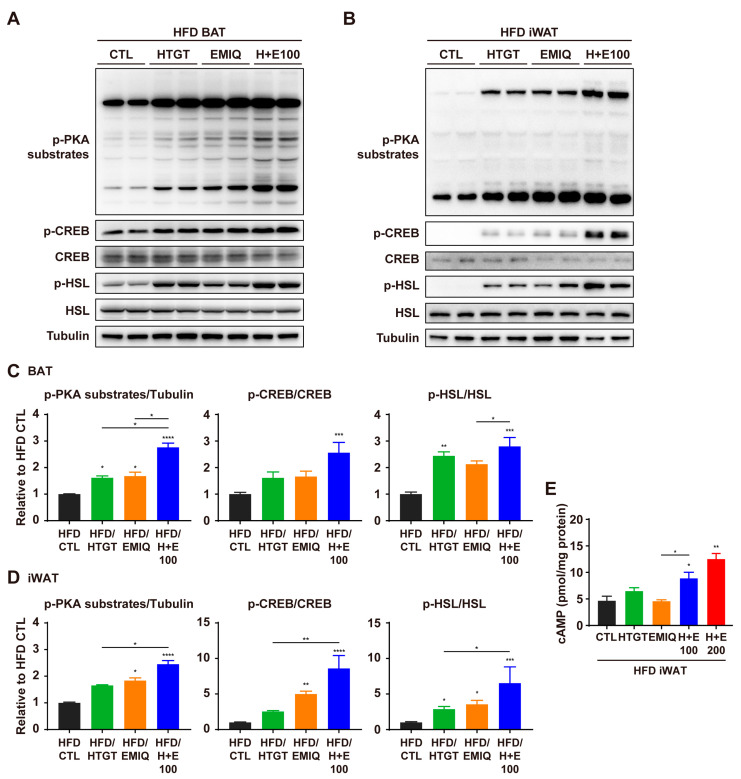
Synergistic effects of co-treatment of HTGT and EMIQ on PKA signaling pathway in HFD-fed mice. (**A**,**B**) Immunoblot analysis of proteins involved in PKA signaling in BAT (**A**) and iWAT (**B**); (**C**,**D**) The quantitative data of protein expression in BAT (**C**) and iWAT (**D**); (**E**) Cyclic adenosine monophosphate (cAMP) levels in iWAT. Statistical significance was assessed using the Kruskal–Wallis test. The data are presented as means ± SEM. *n* = 6. * *p* < 0.05; ** *p* < 0.01; *** *p* < 0.001; **** *p* < 0.0001.

A previous study has reported that the combination of HTGT and EMIQ increased the phosphorylation of HSL in vitro [13], suggesting that the cAMP/PKA signaling pathway may be involved in the observed effects of HTGT and EMIQ. Thus, we hypothesized that the combination of HTGT and EMIQ has a synergism on PKA-dependent lipolysis and mitochondrial metabolism in HFD-fed mice. As expected, the phosphorylation of PKA-downstream proteins was higher following co-treatment, compared to treatment with the individual phytochemicals, in both BAT and iWAT (Figure 5A–D). In addition, we estimated the cAMP levels in iWAT, and found that co-treatment of HTGT and EMIQ significantly increased the cAMP in a dose-dependent manner compared to single treatments (Figure 5E).

**Figure 6:**
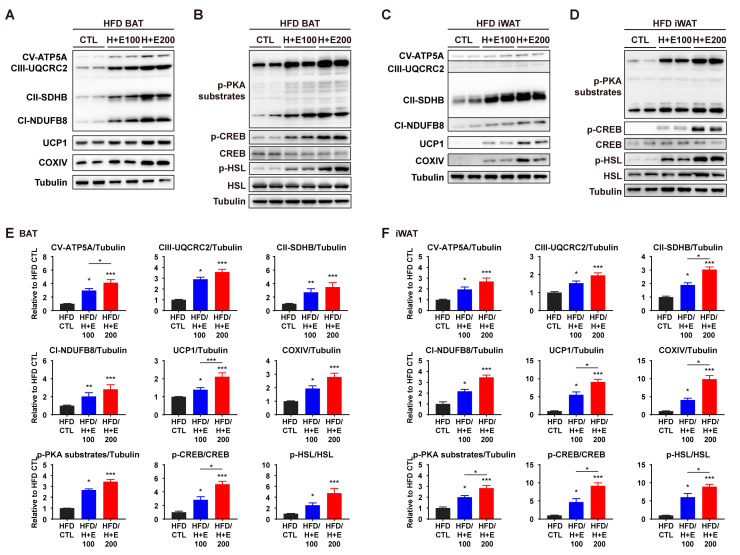
Dose-dependent effects of co-treatment of HTGT and EMIQ on mitochondrial activity and PKA signaling pathway in HFD-fed mice. (**A**–**D**) Immunoblot of proteins involved in mitochondrial activity and PKA signaling pathway in BAT (**A**,**B**) and iWAT (**C**,**D**); (**E**,**F**) The quantitative data of protein expression in BAT (**E**) and iWAT (**F**). Statistical significance was assessed using the Kruskal–Wallis test. The data are presented as means ± SEM. *n* = 6. * *p* < 0.05; ** *p* < 0.01; *** *p* < 0.001.

To determine if the expression levels of these proteins exhibited dose-dependent changes, western blotting was performed on two different doses of co-treatment groups. Consistent with the earlier findings, the high-dose group (200 mg/kg) displayed upregulation of proteins involved in mitochondrial metabolism and PKA signaling compared to the low-dose group (100 mg/kg) (Figure 6A–F). These results support the hypothesis that the combination of HTGT and EMIQ activates PKA signaling, which, in turn, promotes lipolysis and thermogenesis in adipose tissues.

**Figure 7:**
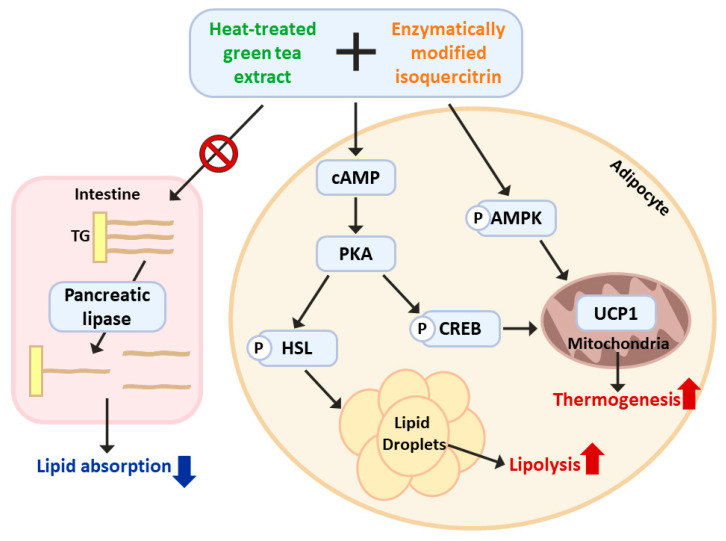
Schematic diagram of the working hypothesis: Molecular mechanisms of HTGT and EMIQ co-treatment in the prevention of obesity and improvement of metabolic health.

Our results revealed that the combined administration of HTGT and EMIQ led to an increase in AMPKα phosphorylation in both BAT and iWAT. Furthermore, we observed a downregulation of p-mTOR levels in iWAT, while no significant changes were observed in BAT, suggesting a depot-specific response of mTOR signaling to the treatment in adipose tissue (Figure S2A,B). To elucidate the role of PKA signaling and AMPK activation in the effects mediated by HTGT and EMIQ, we employed pharmacological inhibitors, specifically H89 (a PKA inhibitor) and Compound C (an AMPK inhibitor). We observed that the combination of HTGT and EMIQ significantly increased the phosphorylation of HSL and AMPK, consistent with our previous findings. Notably, co-treatment with H89 attenuated the effects of the HTGT and EMIQ mixture on HSL phosphorylation, while Compound C abolished the AMPK activation induced by HTGT and EMIQ (Figure S3A,B). These results strongly suggest that the co-treatment exerts its anti-obesity effects through PKA signaling and AMPK activation (Figure 7).

## 4. Discussion

Recent studies have highlighted the potential of adipose tissue browning as a promising approach for anti-obesity therapy. In this context, dietary phytochemicals, such as resveratrol, curcumin, and EGCG, have emerged as notable candidates, demonstrating the ability to induce adipose tissue browning and exert anti-obesity effects [30,31,32].

Combination drug therapy has emerged as a strategy to enhance therapeutic efficacy in metabolic disorders by targeting multiple mechanisms of action and leveraging synergistic effects. By combining different drugs, their complementary actions can lead to improved outcomes and a more comprehensive approach to treatment.

In the current study, we investigated the synergistic anti-obesity effects of combination therapy comprising HTGT and EMIQ. The study observed that co-treatment of HTGT and EMIQ resulted in a dose-dependent reduction in body weight gain and adipocyte hypertrophy in white adipose tissues. During the 8-week high-fat diet (HFD) feeding period, the vehicle control group showed a body weight increase of 15.02 ± 0.8662 g. In contrast, the low-dose of HTGT and EMIQ (HFD/H + E100) group exhibited a lower body weight increase of 7.117 ± 0.2613 g, while the high-dose group (HFD/H + E200) demonstrated an even lower increase of 5.983 ± 0.2725 g (Figure 1). Notably, the combination treatments resulted in significant reductions in body fat mass, increases in lean mass, and reductions in abdominal subcutaneous and visceral fat volumes (Figure 2). Moreover, co-treatment of HTGT and EMIQ improved glucose tolerance and increased energy expenditure. The combination of HTGT and EMIQ exhibited potent inhibition of pancreatic lipase activity with an IC50 value of 46.02 μg/mL, contributing to its anti-obesity effect by enhancing lipid excretion in feces (Figure 3). Furthermore, the combination treatment enhanced mitochondrial activity and promoted thermogenesis in adipose tissues (Figure 4). Moreover, co-treatment of HTGT and EMIQ significantly upregulated cAMP-dependent PKA signaling (Figure 5 and Figure 6), supported by the increase in cAMP levels in WAT (vehicle control: 4.661 ± 0.8584 pmol/mg, low-dose H + E: 8.897 ± 1.148 pmol/mg protein, high-dose H + E: 12.51 ± 1.075 pmol/mg protein).

Green tea catechin promotes lipolysis through PKA-dependent signaling in 3T3-L1 adipocytes. The current study showed that the PKA inhibitor, H89, reduced the catechin-induced phosphorylation of HSL [33]. Previous studies showed that HTGT treatment reduces hepatic fatty acid synthesis, reducing the accumulation of hepatic triacylglycerol and visceral fat in rats [12]. Additionally, various studies have shown that supplementation of quercetin and its glycosides increased the browning markers, including UCP1, in WAT [17,34]. These previous findings reinforce the anti-obesity effects of HTGT and EMIQ, providing further support for the observation in our current study.

It has been previously reported that EMIQ activates AMPK, leading to increased energy expenditure. Furthermore, studies have shown that activation of the AMPK signaling pathway inhibits adipogenesis by reducing mTOR levels [35]. In this study, we observed that the co-treatment of HTGT and EMIQ increased the phosphorylation of AMPK in adipose tissues. In addition, there was a decrease in mTOR phosphorylation in iWAT, but not in BAT (Figure S2). Moreover, the phosphorylation of PKA downstream substrates and AMPK, which were activated by co-treatment of HTGT and EMIQ, were attenuated by the use of a PKA inhibitor and an AMPK inhibitor, respectively (Figure S3). Based on these findings, we hypothesize that the synergistic effect of HTGT and EMIQ arises from the activation of two distinct pathways, namely PKA signaling and AMPK signaling. However, further studies are required to investigate how these independent pathways contribute to the increased beneficial effects observed in obesity prevention. Furthermore, the precise mechanisms underlying the synergism of HTGT and EMIQ remain unclear; thus, additional studies are needed to fully elucidate the molecular mechanisms of how these materials exert synergistic effects.

The development of obesity has been associated with dysregulated epigenetic control, which involves the dysregulation of adipokine secretion such as leptin and adiponectin [36,37]. Growing evidence suggests that bioactive compounds derived from diets can modulate epigenetic mechanisms [38]. Previous studies have explored the effects of green tea and quercetin on chromatin dynamics and epigenetics. For instance, green tea extract prevented HFD-induced up-regulation of miR-335 in epididymal adipose tissue, which is induced by TNFα [39]. Similarly, quercetin and its derivatives induce chromatin remodeling and histone modification at the 5′ regulatory regions of Cebpa and Pparγ in 3T3-L1 preadipocytes [40]. Therefore, further research is warranted to determine whether the co-treatment of HTGT and EMIQ induces change in chromatin and epigenetic mechanisms involved in obesity and metabolic disorders.

While functional foods are typically consumed over extended periods, our previous study only demonstrated the short-term anti-obesity effects of co-administration in mice with HFD-induced obesity [13]. Therefore, this study aimed to investigate the preventive effects of long-term treatment against HFD-induced obesity, which is more relevant to clinical applications. Additionally, the previous study primarily focused on the anti-obesity effects of a single dose of co-administration in vivo. In contrast, this study examined preventive effects against HFD-induced obesity in a synergistic and dose-dependent manner. The experimental results collectively demonstrated that the high dose of the co-treatment group (200 mg/kg) exhibited improved metabolic effects. However, it is crucial to closely monitor potential adverse effects to ensure safety. Furthermore, the translation of these observed effects to human subjects and the determination of an optimal dose remain uncertain. Therefore, further clinical studies or additional research are necessary to establish the efficacy and appropriate dosage for potential clinical use. Additionally, individual variations such as age, gender, and underlying health conditions, should be considered when determining personalized treatment dosages.

## 5. Conclusions

In conclusion, our findings demonstrate that the combination of HTGT and EMIQ exhibits synergistic and dose-dependent preventative anti-obesity effects. This combination holds promise as an approach to obesity prevention and mitigating associated metabolic diseases, making it a potential candidate for the development of dietary supplements. The results of this study provide valuable insights into the utilization of HTGT and EMIQ as dietary supplements for effectively preventing obesity and its related diseases.

## Figures and Tables

**Table 1:** The primer sequences.

Gene	Forward 5′-3′	Reverse 5′-3′
Ppia	GTGGTCTTTGGGAAGGTGAA	TTACAGGACATTGCGAGCAG
Ucp1	TGGCCTCTCAGTGGATGTG	CGTGGTCTCCCAGCATAGAAG

## Data Availability

The data provided in this research are available within the article.

## Supplementary Materials

The following supporting information can be downloaded at: https://www.mdpi.com/article/10.3390/nu15132931/s1, Figure S1: The levels of serum adiponectin and leptin; Figure S2: Effects of co-treatment of HTGT and EMIQ on AMPK/mTOR signaling in adipose tissues; Figure S3: Effects of pharmacological inhibitors of PKA and AMPK on the co-treatment effects of HTGT and EMIQ (H + E).; Table S1: Composition of catechins in green tea and heat-treated green tea extract; Table S2: Primary antibodies used for western blot analysis.

## References

[1] Kopelman P.G. (2000). Obesity as a medical problem. Nature.

[2] González-Castejón M., Rodriguez-Casado A. (2011). Dietary phytochemicals and their potential effects on obesity: A review. Pharmacol. Res..

[3] Blüher M. (2019). Obesity: Global epidemiology and pathogenesis. Nat. Rev. Endocrinol..

[4] Kershaw E.E., Flier J.S. (2004). Adipose Tissue as an Endocrine Organ. J. Clin. Endocrinol. Metab..

[5] Vitali A., Murano I., Zingaretti M.C., Frontini A., Ricquier D., Cinti S. (2012). The adipose organ of obesity-prone C57BL/6J mice is composed of mixed white and brown adipocytes. J. Lipid Res..

[6] Cannon B., Nedergaard J. (2004). Brown adipose tissue: Function and physiological significance. Physiol. Rev..

[7] Sakers A., De Siqueira M.-j.K., Seale P., Villanueva C.J. (2022). Adipose-tissue plasticity in health and disease. Cell.

[8] Lee Y.H., Mottillo E.P., Granneman J.G. (2014). Adipose tissue plasticity from WAT to BAT and in between. Biochim. Biophys Acta.

[9] Yehuda-Shnaidman E., Buehrer B., Pi J., Kumar N., Collins S. (2010). Acute stimulation of white adipocyte respiration by PKA-induced lipolysis. Diabetes.

[10] Mottillo E.P., Bloch A.E., Leff T., Granneman J.G. (2012). Lipolytic products activate peroxisome proliferator-activated receptor (PPAR) α and δ in brown adipocytes to match fatty acid oxidation with supply. J. Biol. Chem..

[11] Ananingsih V.K., Sharma A., Zhou W. (2013). Green tea catechins during food processing and storage: A review on stability and detection. Food Res. Int..

[12] Ikeda I., Hamamoto R., Uzu K., Imaizumi K., Nagao K., Yanagita T., Suzuki Y., Kobayashi M., Kakuda T. (2005). Dietary Gallate Esters of Tea Catechins Reduce Deposition of Visceral Fat, Hepatic Triacylglycerol, and Activities of Hepatic Enzymes Related to Fatty Acid Synthesis in Rats. Biosci. Biotechnol. Biochem..

[13] Im H., Lee J., Kim K., Son Y., Lee Y.-H. (2022). Anti-obesity effects of heat-transformed green tea extract through the activation of adipose tissue thermogenesis. Nutr. Metab..

[14] Valentová K., Vrba J., Bancířová M., Ulrichová J., Křen V. (2014). Isoquercitrin: Pharmacology, toxicology, and metabolism. Food Chem. Toxicol..

[15] Akiyama T., Washino T., Yamada T., Koda T., Maitani T. (2000). Constituents of enzymatically modified isoquercitrin and enzymatically modified rutin (extract). Food Hyg. Saf. Sci. Shokuhin Eiseigaku Zasshi.

[16] Murota K., Matsuda N., Kashino Y., Fujikura Y., Nakamura T., Kato Y., Shimizu R., Okuyama S., Tanaka H., Koda T. (2010). α-Oligoglucosylation of a sugar moiety enhances the bioavailability of quercetin glucosides in humans. Arch. Biochem. Biophys..

[17] Jiang H., Yoshioka Y., Yuan S., Horiuchi Y., Yamashita Y., Croft K.D., Ashida H. (2019). Enzymatically modified isoquercitrin promotes energy metabolism through activating AMPKα in male C57BL/6 mice. Food Funct..

[18] Kim M., Im S., Cho Y.K., Choi C., Son Y., Kwon D., Jung Y.S., Lee Y.H. (2020). Anti-Obesity Effects of Soybean Embryo Extract and Enzymatically-Modified Isoquercitrin. Biomolecules.

[19] Hasumura M., Yasuhara K., Tamura T., Imai T., Mitsumori K., Hirose M. (2004). Evaluation of the toxicity of enzymatically decomposed rutin with 13-weeks dietary administration to Wistar rats. Food Chem. Toxicol..

[20] Judex S., Luu Y.K., Ozcivici E., Adler B., Lublinsky S., Rubin C.T. (2010). Quantification of adiposity in small rodents using micro-CT. Methods.

[21] Luu Y.K., Lublinsky S., Ozcivici E., Capilla E., Pessin J.E., Rubin C.T., Judex S. (2009). In vivo quantification of subcutaneous and visceral adiposity by micro-computed tomography in a small animal model. Med. Eng. Phys..

[22] Choi C., Song H.D., Son Y., Cho Y.K., Ahn S.Y., Jung Y.S., Yoon Y.C., Kwon S.W., Lee Y.H. (2020). Epigallocatechin-3-Gallate Reduces Visceral Adiposity Partly through the Regulation of Beclin1-Dependent Autophagy in White Adipose Tissues. Nutrients.

[23] Ayala J.E., Samuel V.T., Morton G.J., Obici S., Croniger C.M., Shulman G.I., Wasserman D.H., McGuinness O.P., NIH Mouse Metabolic Phenotyping Center Consortium (2010). Standard operating procedures for describing and performing metabolic tests of glucose homeostasis in mice. Dis. Model. Mech..

[24] Seo D.-B., Jeong H.W., Kim Y.-J., Kim S., Kim J., Lee J.H., Joo K., Choi J.K., Shin S.S., Lee S.-J. (2017). Fermented green tea extract exhibits hypolipidaemic effects through the inhibition of pancreatic lipase and promotion of energy expenditure. Br. J. Nutr..

[25] Kraus D., Yang Q., Kahn B.B. (2015). Lipid Extraction from Mouse Feces. Bio. Protoc..

[26] Cho Y.K., Yoon Y.C., Im H., Son Y., Kim M., Saha A., Choi C., Lee J., Lee S., Kim J.H. (2022). Adipocyte lysoplasmalogenase TMEM86A regulates plasmalogen homeostasis and protein kinase A-dependent energy metabolism. Nat. Commun..

[27] Ikeda I., Tsuda K., Suzuki Y., Kobayashi M., Unno T., Tomoyori H., Goto H., Kawata Y., Imaizumi K., Nozawa A. (2005). Tea catechins with a galloyl moiety suppress postprandial hypertriacylglycerolemia by delaying lymphatic transport of dietary fat in rats. J. Nutr..

[28] Zhou J.-F., Wang W.-J., Yin Z.-P., Zheng G.-D., Chen J.-G., Li J.-E., Chen L.-L., Zhang Q.-F. (2021). Quercetin is a promising pancreatic lipase inhibitor in reducing fat absorption in vivo. Food Biosci..

[29] Lee J.H., Park A., Oh K.-J., Lee S.C., Kim W.K., Bae K.-H. (2019). The Role of Adipose Tissue Mitochondria: Regulation of Mitochondrial Function for the Treatment of Metabolic Diseases. Int. J. Mol. Sci..

[30] Alberdi G., Rodríguez V.M., Miranda J., Macarulla M.T., Churruca I., Portillo M.P. (2013). Thermogenesis is involved in the body-fat lowering effects of resveratrol in rats. Food Chem..

[31] Lone J., Choi J.H., Kim S.W., Yun J.W. (2016). Curcumin induces brown fat-like phenotype in 3T3-L1 and primary white adipocytes. J. Nutr. Biochem..

[32] Lee M.S., Shin Y., Jung S., Kim Y. (2017). Effects of epigallocatechin-3-gallate on thermogenesis and mitochondrial biogenesis in brown adipose tissues of diet-induced obese mice. Food Nutr. Res..

[33] Chen S., Osaki N., Shimotoyodome A. (2015). Green tea catechins enhance norepinephrine-induced lipolysis via a protein kinase A-dependent pathway in adipocytes. Biochem. Biophys. Res. Commun..

[34] Jiang H., Horiuchi Y., Hironao K.Y., Kitakaze T., Yamashita Y., Ashida H. (2020). Prevention effect of quercetin and its glycosides on obesity and hyperglycemia through activating AMPKα in high-fat diet-fed ICR mice. J. Clin. Biochem. Nutr..

[35] Ye Y., Liu H., Zhang F., Hu F. (2019). mTOR signaling in Brown and Beige adipocytes: Implications for thermogenesis and obesity. Nutr. Metab..

[36] Peng Y., Yu S., Li H., Xiang H., Peng J., Jiang S. (2014). MicroRNAs: Emerging roles in adipogenesis and obesity. Cell. Signal..

[37] Houde A.-A., Légaré C., Biron S., Lescelleur O., Biertho L., Marceau S., Tchernof A., Vohl M.-C., Hivert M.-F., Bouchard L. (2015). Leptin and adiponectin DNA methylation levels in adipose tissues and blood cells are associated with BMI, waist girth and LDL-cholesterol levels in severely obese men and women. BMC Med. Genet..

[38] Vahid F., Zand H., Nosrat–Mirshekarlou E., Najafi R., Hekmatdoost A. (2015). The role dietary of bioactive compounds on the regulation of histone acetylases and deacetylases: A review. Gene.

[39] Otton R., Bolin A.P., Ferreira L.T., Marinovic M.P., Rocha A.L.S., Mori M.A. (2018). Polyphenol-rich green tea extract improves adipose tissue metabolism by down-regulating miR-335 expression and mitigating insulin resistance and inflammation. J. Nutr. Biochem..

[40] Nettore I.C., Rocca C., Mancino G., Albano L., Amelio D., Grande F., Puoci F., Pasqua T., Desiderio S., Mazza R. (2019). Quercetin and its derivative Q2 modulate chromatin dynamics in adipogenesis and Q2 prevents obesity and metabolic disorders in rats. J. Nutr. Biochem..

