# Supplementary material for: Synergistic Effects of Heat-Treated Green Tea Extract and Enzymatically-Modified Isoquercitrin in Preventing Obesity
Source: Nutrients. 2023 Jun 28;15(13):2931. doi: 10.3390/nu15132931 (PMC10346566; doi:10.3390/nu15132931)
Supplement: Supplementary file 1 [file nutrients-15-02931-s001.zip › nutrients-2446796-supplementary.pdf]

Supplementary

Table S1. Composition of catechins in green tea extract and heat-treated green tea extract.

Catechin	Green tea extract	Heat-treated green tea extract
**EGCG**	16.27	5.2
**GCG**	1.19	5.6
**EGC**	9.99	3.1
**GC**	0.77	4.8
**ECG**	3.19	1.3
**CG**	0.04	1.2
**EC**	3.5	0.95
**C**	0.63	1.57
**Total**	35.57	23.97

Table S2. Primary antibodies used for western blot analysis.

Antibody	Host	Manufacturer	Catalog #	Dilution
Total OXPHOS	Mouse	Abcam	Ab110413	1:1000
UCP1	Rabbit	Alpha Diagnostic International	UCP11-A	1:1000
COXIV	Rabbit	Cell Signaling	4850	1:1000
Phospho-PKA substrate	Rabbit	Cell signaling	9621	1:1000
Phospho-CREB	Rabbit	Cell signaling	9198	1:1000
CREB	Rabbit	Cell Signaling	9197	1:1000
Phospho-HSL	Rabbit	Cell Signaling	45804	1:1000
HSL	Rabbit	Cell Signaling	4107	1:1000
Phospho-AMPKα	Rabbit	Cell Signaling	2531S	1:1000
AMPKα	Rabbit	Cell Signaling	2532S	1:1000
Phospho-mTOR(Ser2448)	Rabbit	Cell Signaling	5536S	1:1000
mTOR	Rabbit	Cell Signaling	2983P	1:1000
α/β-Tubulin	Rabbit	Cell Signaling	2148	1:1000

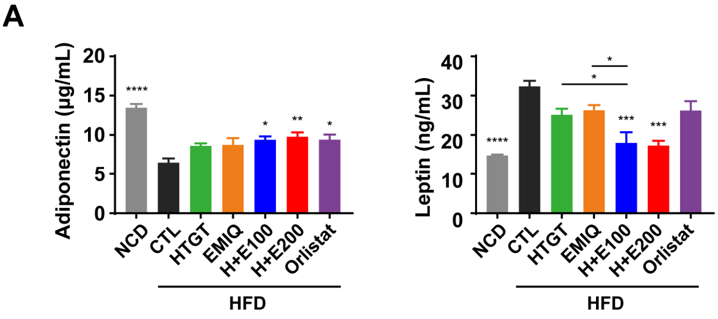

Figure S1. The levels of serum adiponectin and leptin. Statistical significance was assessed using Kruskal-Wallis test. The data are presented as means ± standard error of the mean (SEM). n=6. * $p < 0.05$; ** $p < 0.01$; *** $p < 0.001$; **** $p < 0.0001$.

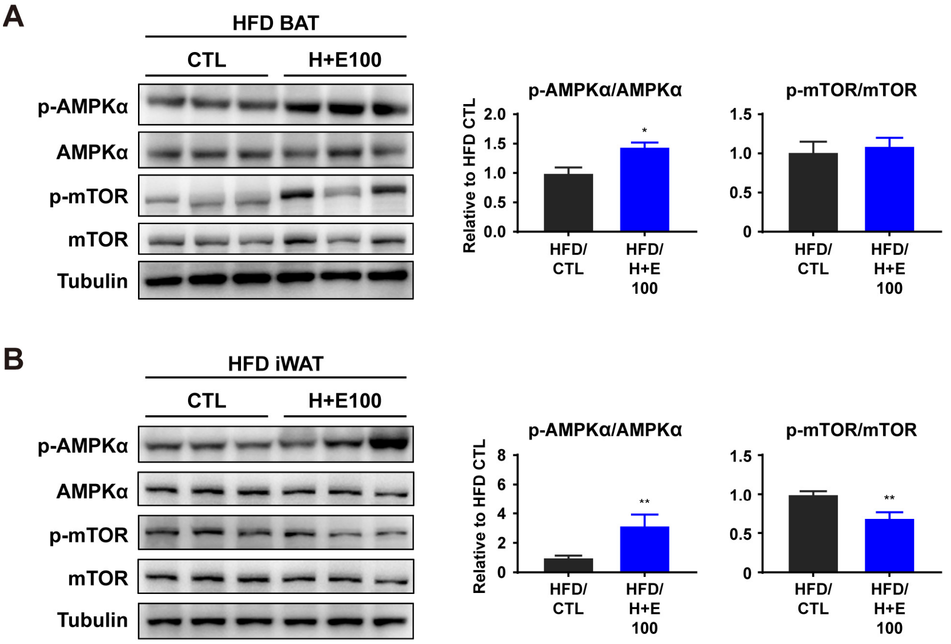

Figure S2. Effects of co-treatment of HTGT and EMIQ on AMPKα and mTOR signaling in adipose tissues. (A, B) Immunoblot analysis and quantification of proteins involved in phosphorylation of AMPKα (Thr258 and Ser485 for α1, Ser491 for α2) and mTOR (Ser2448) in BAT (A) and iWAT (B). Statistical significance was determined using the Mann-Whitney test. The data are presented as means ± SEM. *n*=6. * $p < 0.05$; ** $p < 0.01$.

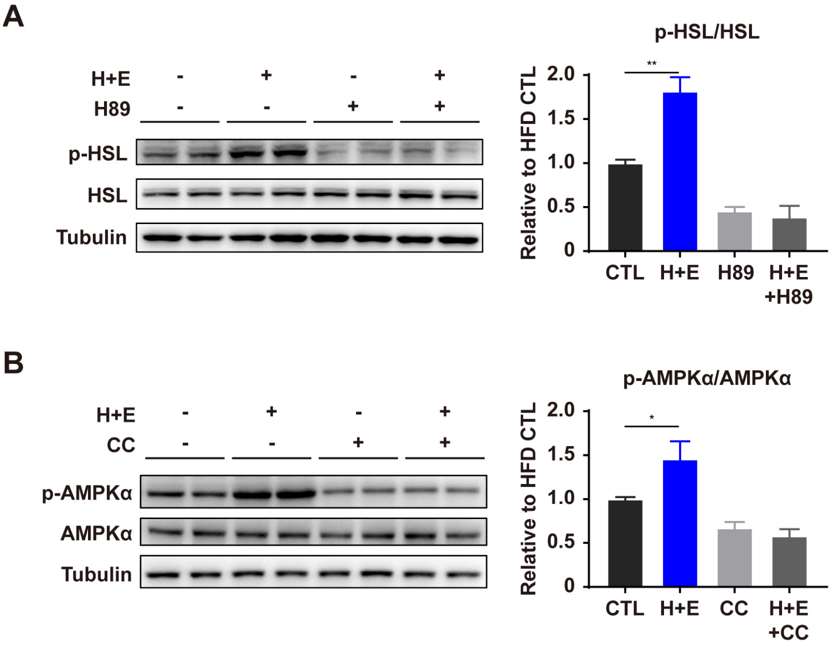

Figure S3. Effects of pharmacological inhibitors of PKA and AMPK on the co-treatment effects of HTGT and EMIQ (H+E). (A) Protein expression levels of p-HSL and HSL in adipocytes treated with H+E (100 μg/mL) and H89 (PKA inhibitor, 50 μM) for 24h. (B) Protein expression levels of p-AMPKα and AMPKα in adipocytes treated with H+E (100 μg/mL) and Compound C (CC, AMPK inhibitor, 10 μM). Statistical significance was assessed using the Kruskal-Wallis test. The data are presented as means ± SEM. n=4. * $p < 0.05$; ** $p < 0.01$.
